# Perspectives of Policy Makers and Service Users Concerning the Implementation of eHealth in Sweden: Interview Study

**DOI:** 10.2196/28870

**Published:** 2022-01-28

**Authors:** Margit Neher, Annette Nygårdh, Anders Broström, Johan Lundgren, Peter Johansson

**Affiliations:** 1 Department of Rehabilitation School of Health and Welfare Jönköping University Jönköping Sweden; 2 Department of Nursing Sciences School of Health and Welfare Jönköping University Jönköping Sweden; 3 Department of Clinical Neurophysiology Linköping University Hospital Linköping Sweden; 4 Department of Health, Medicine and Caring Sciences Linköping University Linköping Sweden

**Keywords:** clients, computer-assisted therapy, consultation telehealth, decision-makers, implementation, patients, politicians, qualitative methods, remote, mobile phone

## Abstract

**Background:**

Increasing life spans of populations and a growing demand for more advanced care make effective and cost-efficient provision of health care necessary. eHealth technology is often proposed, although research on barriers to and facilitators of the implementation of eHealth technology is still scarce and fragmented.

**Objective:**

The aim of this study is to explore the perceptions concerning barriers to and facilitators of the implementation of eHealth among policy makers and service users and explore the ways in which their perceptions converge and differ.

**Methods:**

This study used interview data from policy makers at different levels of health care (n=7) and service users enrolled in eHealth interventions (n=25). The analysis included separate qualitative content analyses for the 2 groups and then a second qualitative content analysis to explore differences and commonalities.

**Results:**

Implementation barriers perceived by policy makers were that not all service users benefit from eHealth and that there is uncertainty about the impact of eHealth on the work of health care professionals. Policy makers also perceived political decision-making as complex; this included problems related to provision of technical infrastructure and lack of extra resources for health care digitalization. Facilitators were policy makers’ conviction that eHealth is what citizens want, their belief in eHealth solutions as beneficial for health care practice, and their belief in the importance of health care digitalization. Barriers for service users comprised capability limitations and varied preferences of service users and a mismatch of technology with user needs, lack of data protection, and their perception of eHealth as being more time consuming. Facilitators for service users were eHealth technology design and match with their skill set, personal feedback and staff support, a sense of privacy, a credible sender, and flexible use of time.There were several commonalities between the 2 stakeholder groups. Facilitators for both groups were the strong impetus toward technology adoption in society and expectations of time flexibility. Both groups perceived barriers in the difficulties of tailoring eHealth, and both groups expressed uncertainty about the care burden distribution. There were also differences: policy makers perceived that their decision-making was very complex and that resources for implementation were limited. Service users highlighted their need to feel that their digital data were protected and that they needed to trust the eHealth *sender*.

**Conclusions:**

Perceptions about barriers to and facilitators of eHealth implementation varied among stakeholders in different parts of the health care system. The study points to the need to reach an enhanced mutual understanding of priorities and overcome challenges at both the micro and macro levels of the health care system. More well-balanced decisions at the policy-maker level may lead to more effective and sustainable development and future implementation of eHealth.

## Introduction

### Background

Health care systems around the world are struggling to satisfy the health care needs of their populations. Because of the increasing population life span and the growing demand for more advanced care, the need for effective and cost-efficient provision of health care is a matter of national concern for many nations [1]. eHealth technology is often proposed as a promising way forward for better and more cost-efficient health care solutions. This *umbrella term*, which includes many different digital health care solutions such as telemedicine and the use of mobile messaging devices, has become a buzzword in many countries and is defined in the World Health Assembly resolution WHA58.28 (2005) as “the cost-effective and secure use of ICT in support of health and health-related fields” [2]. Many European health care systems see a proliferation in eHealth technologies, and policy decisions to implement them seem to be made despite the scarcity of convincing research data concerning both claims of cost-effectiveness and their real impact on the quality and safety of health care [3,4]. For example, in Sweden, the implementation of eHealth technologies has been identified as an important strategy toward the future quality provision of population health care. eHealth technology in different forms has been introduced in many different Swedish health care settings, with the hope of better quality of care, better patient empowerment in care, and lower health care costs [5]. At the national level, ambitious political goals have been set to become world leaders in using the opportunities offered by eHealth by the year 2025 [5].

Research on barriers to and facilitators of the implementation of eHealth technology is still scarce and fragmented; however, some reviews have collated what is currently known [6,7]. Implementation was found to have a multilevel complexity: the technology, individual health care providers, inner and outer settings, and process of implementation [7]. Greenhalgh et al [8] constructed an empirically based framework that described implementation complexity in 7 domains: the condition or illness of the patient, the technology, the value proposition, the adopters, the organization, the wider system, and the embedding and adaptation of the technology over time.

Stakeholders in different parts of a health care system may have differing inherent perspectives on key system functions in this complexity, depending on the role they have in the system. Glouberman and Mintzberg [9] observed that when managing changes, barriers need to be broken down between different perspectives on care (*curing*, *caring*, *costs*, and *community*) so that the resources of the overall system can be allocated more effectively. To navigate toward a responsible and sustainable future use of eHealth technology, it will be necessary to take on the challenge of understanding implementation complexity. This includes understanding the views of both the *back-end* stakeholders in an implementer role (the policy makers) and the *front-end* stakeholders in an end user role (the service users). Although several recent studies have identified factors that influence the perceptions of stakeholders in different populations [10-12], research on the experiences of stakeholders in a policy role is scarce, as is knowledge about the overlap between policy and public (service user) perspectives. The viewpoints of policy makers are seldom recorded in scientific publications despite their important role in the *outer context* of implementation [13-15].

### Objectives

The aim of this study is to explore the perceptions concerning the barriers to and facilitators of the implementation of eHealth as perceived by policy makers and service users, and explore the ways in which their perceptions converge and differ. Ultimately, the study may contribute new knowledge to more effectively co-design the future implementation of digital health care technology.

## Methods

### Design

This study used a qualitative design in 2 steps, including interviews with 7 policy makers and 25 service users from the southeast of Sweden.

### Participants and Interviews

The study includes primary data from 7 persons in a policy-making role and secondary data from 25 persons who had personal experience in an eHealth intervention.

The policy makers, employed in 4 different county councils in Southeast Sweden, were recruited by phone or email at the beginning of 2018. During a recruitment period of several months, a total of 23 persons representing different levels of policy makers in health care were contacted. Of these 23 persons, 14 (61%) declined to participate because of full agendas, and 2 (9%) did not answer the invitation.

The service user data were retrieved from 3 different eHealth intervention effectiveness studies [16-18]. Participants were provided written and oral information concerning the objectives and procedures of the study. The document informed the study participants that participation was voluntary and that they could stop at any moment with no reason given. All participants gave their informed consent.

Interviews with policy makers were conducted either face to face or by telephone, depending on their choice. The interview guide for policy makers was developed from the Swedish national eHealth policy document, with which participants were familiar [5]. The participants were asked to describe their perceptions and experiences with eHealth technology in their stakeholder role, their perceptions of the pros and cons of eHealth technology, their knowledge of policy documents related to eHealth technology, and their perceptions of factors influencing the implementation of eHealth in clinical practice. Participants were interviewed individually either at their workplace or by telephone (according to their preference). Interviews lasted for a duration of 43 minutes to 63 minutes.

Service users were interviewed via telephone. In HeaDING (Heart Failure and Depression Using Internet Based–Cognitive Behavioural Therapy), service users were interviewed post intervention in 2015 [16]. All service users (n=27, with a median age of 69 years) who had been enrolled in internet-based cognitive behavioral therapy (iCBT) were invited to the interview study; of the 27 patients, 13 (48%) agreed to participate; of the 13 patients, 8 (51%) were men, and 5 (39%) were women. Interviews from 5 women and 5 men were used for the analysis in this study. Service users in DOHART (a research study concerning iCBT for persons with heart disease and depressive symptoms) [17] were interviewed post intervention between January and June 2017. A total of 35 service users with a mean age of 62 years were invited; of the 35 service users, 20 (57%) accepted to be interviewed; of the 20 participants, 11 (55%) were men, and 9 (45%) were women. In both HeaDING and DOHART studies, service users were included if they were receiving cardiovascular disease (CVD) treatment according to the current guidelines for heart failure, coronary artery disease, and atrial fibrillation and had at least mild depression (Patient Health Questionnaire ≥5 points). Interviews from 5 men and 5 women were used in this study. The service users in Heart And Insomnia Treatment Through Internet. (a research study concerning iCBT treatment for insomnia in patients with CVD) were interviewed before the intervention (late 2017) [18]. Service users had primary CVD as defined by diagnostic codes from the International Classification of Disease and sleep disorders. Diagnoses included myocardial infarction, angina pectoris, heart failure, atrial fibrillation or flutter, or arrhythmia. Service users were recruited from 6 primary care centers to participate in the randomized controlled trial. All participants in the intervention group (n=24, mean age 72 years) who had started their treatment were invited to participate in the interviews. As the study was ongoing at the time of this study, only 5 interviews (n=3, 60% men and n=2, 40% women) had been conducted, all of which were used for this study.

The primary focus of these interviews was on participants’ experiences with the specific eHealth intervention in which they were enrolled; the focus on their general perceptions of factors influencing the implementation of eHealth in clinical care was secondary. Questions could concern their general perceptions of the applicability of the eHealth intervention to other users, the skills necessary for using the eHealth intervention, and the pros and cons of eHealth. The quality of the data varied from interview to interview. Where the service user interviews had relevant data for the purpose of this study, excerpts were analyzed. Some interviews were relevant in their entirety. Therefore, there was a wide range of interview duration (15-100 minutes).

Interviewers had clinical or research experience in cardiac nursing (service user interviews) or clinical and research experience in implementation research and health care (policy maker interviews), and all were experienced interviewers. All interviews were audio-recorded and transcribed verbatim.

### Data Analysis

Implementation is the conduction, execution, or practice of a plan, a method, or any design, idea, model, specification, standard, or policy for doing something. Implementation science is “the scientific study of methods to promote the systematic uptake of research findings and other EBPs into routine practice, and, hence, to improve the quality and effectiveness of health services” [19]. The term *barrier* is used in implementation science to denote the challenges that may occur during the process of implementation and that may limit the effectiveness of the implementation strategy used. The term *facilitator* is used to denote a factor (condition) that may make implementing something easier. The analysis was performed in 2 steps. The first analysis (assorted analysis) explored the perceptions of each participant group separately, and the second analysis (supplementary analysis) explored aspects that needed to be understood further [20].

In the first step, the aim was to explore perceptions concerning barriers to and facilitators of the implementation of eHealth from the separate perspectives of policy makers on the one hand and service users on the other.

The data from policy makers and the data from service users were initially analyzed separately in each group by MN and JL in a process called *assorted analysis* [20] and adhered to the procedure of inductive qualitative content analysis as described by Hsieh and Shannon [21]. MN and JL read and reread the data several times using an inductive approach. They identified meaning units comprising words or sentences and, after subjecting these to a critical analysis, combined the content into separate sets of subcategories and categories. Barriers to and facilitators of the implementation of eHealth emerged in each analysis; these were the main categories within each group. A number of nonoverlapping and mutually excluding subcategories also emerged within the main categories for each group. To support trustworthiness, the results from these analyses were thereafter iteratively discussed in the wider author group until consensus was reached. The analyses were assisted by a computer software (NVivo 11 [QSR International]).

In the second step, a *supplementary analysis* was undertaken to explore aspects of the data that were not fully addressed in the first analysis [20]. This analysis (an analysis between groups), in which the perceptions of the 2 groups were compared and contrasted to explore differences and commonalities between policy makers and service users, also followed the procedure of inductive qualitative content analysis [21]. JL and MN undertook the initial analysis by iteratively examining the qualitative data in the subcategories representing the barriers and facilitators in each group of interviews, finding the points where they converged and differed. When necessary, the original data from the interviews with service users and policy makers were consulted to check for consistency and accuracy. To support trustworthiness, the results from these analyses were then iteratively discussed in the wider author group until consensus was reached.

### Ethical Considerations

The study was performed in accordance with the Swedish ethical law and the Declaration of Helsinki. The regional ethics board approved the study (Dnr 2016/72-31; Dnr 2015/258-31, 2017/378-32; Dnr 2011.166/31).

## Results

### Overview

The participants in the policy maker group had a variety of roles ranging from elected politicians with a political responsibility for health care portfolios at the regional level (representing 3 different political parties) to administrators in a more operational capacity at regional or local levels. All had an explicit mission to implement information technology in health care. The participants (5/7, 71% women and 2/7, 29% men) were aged between 40 and 59 years and had work experience between 4 and 20 years in their present role. The participants in the service user group (9/25, 36% women and 16/25, 64% men) were aged 39 to 83 years and varied in educational background. Participants had various heart diseases: ischemic heart disease, heart failure, arrhythmia, or combinations thereof. All service users had an experience with eHealth, either having been introduced to, participating in, or having participated in an eHealth intervention.

### Policy Makers

#### Overview

For policy makers, 5 barriers and 3 facilitators for the implementation of eHealth were found (Table 1).

**Table 1:** Main categories and subcategories with quotes from the qualitative content analysis (policy makers’ perceptions of barriers to and facilitators of the implementation of eHealth interventions in health care).^a^

Subcategories			Quotes
**Barriers**			
	Belief that not all service users will benefit from eHealth	“[eHealth technology]...will suit some people’s needs, but different people need different things...I really think you should see it as complementary and not instead of [standard care]” [Policy maker 2]	
	Uncertainty about the work consequences for health care professionals	“We have staff working with the technical coordination...providing access and such...But about the human development...How you change the ways [health care] staff do their work...To be honest, I think we haven’t been thinking about that a lot” [Policy maker 7]	
	Fear of problems with providing technical infrastructure	“We have to have a working internet in all parts of our region...If you can’t get a sufficiently strong connection, that is going to be a problem” [Policy maker 2]	
	Perceived complexity of political decision-making	“There are at least 60 projects on-going [in our region] at the moment...We don’t have an accepted way to make decisions: we can’t compare different proposals, and we can’t prioritize...we have no evaluation tool...We can’t decide what is most important...is it what politicians think is best, or is it about how it affects our economy?...That’s why it becomes a matter for everybody and also for nobody, because there isn’t an obvious person or group who is responsible” [Policy maker 1]	
	Lack of extra resources for eHealth implementation	“I think about ‘effect’ from an economic perspective...all our practices are driven by economic considerations...It should be a smart solution...So if you use a technical solution, it should eradicate another cost” [Policy maker 3]	
**Facilitators**			
	Policy makers’ conviction that eHealth is what citizens want	“For us in politics, the citizen perspective is the most important. To be able to access your own medical file, to sit at home and receive cognitive behavioral therapy. To be able to provide a chance to participate in the care process...it makes patients more participatory in their care” [Policy maker 4]	
	Policy makers’ belief in eHealth solutions as beneficial for health care practice	“We expect to be more effective, and hope to use the time we gain to help more afflicted patients” [Policy maker 1]	
	Policy makers’ belief in the importance of working toward eHealth implementation in their region	“Digitalization is part of our political mission. Our regional planning has been hampered because we are governed by a political minority which has made decision-making weak, so we have been a bit left behind. But my conviction is that this issue should be taken up to the highest political level...We have to show that we are in the game!” [Policy maker 3]	

^a^Numbers in parentheses are participant codes.

#### Barriers Perceived by Policy Makers

##### Belief That Not All Service Users Benefit From eHealth

Although policy makers expressed very positive views about eHealth technology, some also discussed situations in which eHealth technology would be less beneficial: when professionals meet service users for the first time, when they prescribe psychiatric medication or assess the need for sick-leave, or when they treat the young or those service users with complex problems. Some service user characteristics (such as age and condition) were also described as barriers to eHealth technology; however, 1 policy maker thought that service users being uncomfortable with technology was the most important factor.

##### Uncertainty About the Work Consequences for Health Care Professionals

The process of changes in the direction of digitalization was described as uneven, as different work units were perceived to be different in their way of tackling the issue and getting different support in the change process. A policy maker described that the changes that need to happen when introducing a new way of working in the clinic were not very well-developed and that implementing change was difficult.

Policy makers perceived that much effort was usually put into the technical implementation, whereas the development of the workforce was left to the work unit in question. Policy makers were uncertain about whether the planning for change and implementing eHealth technology in practice meant more work instead of less for work units. They perceived that staff could experience an uncertainty about the negative consequences of digitalization for their own employment and thought that staff needed better support for learning about technical solutions in the workplace.

##### Fear of Problems With Providing Technical Infrastructure

The incompatibility of the technical systems and differences in the *language* that systems use was perceived to lead to unnecessary difficulties in information sharing. Data sharing between health care systems was seen as a top priority by several participants, although some also raised the issue of data security.

Policy makers expressed that an important implementation barrier was a lack of knowledge about technical solutions and their real potential applicability in health care. They had many proposals about novel technical eHealth solutions flooding their workday and experienced difficulty managing the need to make implementation decisions in an informed and systematic way. There was uncertainty among policy makers about the organizational chain of responsibility for implementing the new technologies, the different actors concerned in the political system and the health care system, and how roles overlap and differ. As laws and regulations lag behind the current rapid technical development, there was a feeling of not being supported in decision-making (eg, when considering if and how to limit *internet doctors* from inappropriately diverting health care resources).

##### Lack of Extra Resources for eHealth Implementation

Despite the strong political impetus to implement eHealth solutions, policy makers perceived that no extra resources were available in the health care system for eHealth implementation. They perceived that implementation decisions at both the clinical and political levels were influenced by the lack of economical and personal resources, as they saw cost–benefit calculations as a key factor for implementation. They also found that decision-making in their political role was difficult; both the needs of the population and the solutions offered by technology companies were many and multifaceted. They found it hard to prioritize among solutions, as it was not always clear what the costs and gains would be in different cases. Policy makers expressed that they would prefer not to leave the development of digital tools exclusively to commercial agents with a profit agenda or to researchers with limited resources. They reasoned that extra resources should be allocated to implement technology in clinical everyday care and that public funds should engage more actively in supporting innovation, as resources are necessary to design and develop good digital products for health care use.

#### Facilitators Perceived by Policy Makers

The arguments that policy makers used to describe the benefits of eHealth solutions were many, and all policy makers extolled the advantages of digital care solutions for patients, staff, and society at large.

##### Policy Makers’ Conviction That eHealth is What Citizens Want

Policy makers stressed that facilitating the accessibility to care for service users was an important driver for their work. Policy makers perceived that providing citizens with the opportunity to book their health care visits themselves, order their medication, and check their electronic health records on the web would guide health care to become more patient centered. Many perceived that citizens expect society to provide them with access to internet for professional examinations, treatment, and health monitoring, with the benefits of less traveling.

##### Policy Makers’ Belief in eHealth Solutions as Beneficial for Health Care Practice

Perceptions were that eHealth technology could lead to advantages for staff by providing decision support. eHealth could lead to administrative effectiveness and better workflow; as more patients and families (in theory) managed their own health administration, staff could cater to those most in need of personal service.

##### Policy Makers’ Belief in the Importance of Working Toward eHealth Implementation in Their Region

Some policy makers expressed that they saw the necessity to take an active role in politics and fulfill their ambition to push their region to the frontline of health care digitalization and implementation of eHealth. They expressed a wish to be among *the best*. In particular, building infrastructure was seen by policy makers as a crucial part of their political role, as populations in rural regions need broadband and other facilities to access eHealth programs. To reach that goal, policy makers saw a need to work together at the regional and local levels, involve service users, and collaborate with commercial companies and universities.

### Service Users

#### Overview

For service users, 5 barriers to and 6 facilitators of the implementation of eHealth were found (Table 2).

**Table 2:** Main categories and subcategories with quotes from the qualitative content analysis (service users´ perceptions of barriers to and facilitators of the implementation of eHealth in health care).^a^

Subcategories		Quotes
**Barriers**		
	Limitations in the capability of the service user	“You know, for me it was all this digitalisation. I had been better off if I could write on a paper and send it in. I thought it was rather complicated” [Service user 3]
	eHealth is not always what the individual service user wants	“Yeah, when it comes to healthcare I think that you prefer a personal contact. Or so I think” [Service user 4]
	eHealth is perceived as time consuming for the service user	“This a darn lot of time, for the patient, I mean it would take a lot less time if one had an appointment with someone and was there talking for an hour compared to reading a lot and fill out forms and find out answers and write answers” [Service user 3]
	Mismatch of technology with service user needs	“Overall...Yes, I had, I must say had expected something else” [Service user 25]
	Perceived lack of data protection	“I’m not happy about that at all...it’s just a matter of how good a hacker you are...” [Service user 5]
**Facilitators**		
	User-friendly design	“And even if you forget some things as time passed, you could always go back and refresh your memory. So that’s an advantage” [Service user 22]
	Matches skill set of service user	“I had an advantage in that I’m quite structured and used to work with structured materials on the computer” [Service user 22]
	Provides a sense of privacy	“First you had a pass-word and then a single use code, that made me feel rather secure because they [designers of the web-platform] had thought about it” [Service user 2]
	Personal feedback and staff support	“I had rather long questions and I got good answers and good feedback, I really appreciated that and without that it wouldn’t work. It was a necessary and good complement [to the iCBT^b^ program]” [Service user 1]
	Flexible use of time	“If the alternative had been personal meetings, so that you had appointments a number of times, then naturally this [iCBT-program] has clear advantage timewise, because I have rather fully-booked work day, and I don’t have to set more time aside for it” [Service user 26]
	A credible sender	“I think this type of program, this type of treatment or healthcare, it’s totally ok for me because you have a human that you can call and talk to, otherwise it wouldn’t work” [Service user 3]

^a^Numbers in parentheses are participant codes.

^b^iCBT: internet-based cognitive behavioral therapy.

#### Barriers Perceived by Service Users

##### Limitations in the Capability of the Service User

Service users perceived that their health status could limit their ability to use eHealth, for example, because of memory problems. Some service users thought that other service users might hesitate to use digital technology in general and not only described technical problems with the digital solution itself but also the unfamiliarity with computer-based activity, for example, developing a smooth workflow on the web-based platform. They also found that the log-in process was complicated and would have preferred more automated feedback from the platform.

##### eHealth Is Not Always What the Individual Service User Wants

A feeling of being *tied to their computer* was perceived when working with the iCBT program. Service users could perceive that communication in writing with a health care professional was unfamiliar and sometimes uncomfortable for them, and those service users who were not used to reading long texts on screen expressed that they preferred to read from printouts.

Service users described the lack of face-to-face or direct audiovisual communication with the therapist as a barrier, as they perceived that communication in writing sometimes meant missing nuances in the communication. Some service users preferred traditional health care with face-to-face appointments and participation in support groups and therefore perceived eHealth as the second-best alternative.

##### eHealth Is Perceived as Time Consuming for the Service User

Although appreciative of the opportunity to use the eHealth technology when it suited them, service users would have liked to continue being able to contact health care by telephone. As this was perceived as increasingly difficult, they felt obligated to use digital solutions, which they described as complicated and time consuming. Some service users also felt that they had to take more responsibility for their treatment compared with traditional face-to-face treatment, for example, with weekly appointments. This was described as a barrier, especially for patients who experienced that their family or work situation required much time and attention.

##### Mismatch of Technology With Service User Needs

Service users described that they thought that the eHealth interventions in which they had participated did not match their health needs, that the intervention had not been provided at the right time, or that the content of the intervention did not meet their expectations or because it was not sufficiently tailored to their personal needs.

##### Perceived Lack of Data Protection

A barrier could also be uncertainty about personal integrity and information security. Service users who experienced uncertainty about what information could be extracted from different types of devices and telemonitoring expressed rather strong feelings, such as fear.

#### Facilitators Perceived by Service Users

##### User-Friendly Design

Service users perceived that a user-friendly design was important for their use of the eHealth intervention. An appropriate level of complexity in the program texts and communication processes (such as the option of ticking simple yes or no questions) was a feature. Although some service users described it as hard to write about health problems, some also described that the program relied on written communication and not real-time communication, as this meant that they had more time to consider how to communicate with health care staff in addressing sensitive topics. Another feature service users appreciated was the easy availability of the previous parts of the program.

##### Matches Skill Set of Service User

The service users in this study described themselves as relatively regular users of computers and digital services and perceived these experiences as facilitators of the use of an eHealth intervention. Specifically, previous experiences of, for example, working with texts, was perceived as a success factor in the intervention in which they were enrolled, as the iCBT program demanded a capacity for advanced reading and writing. For managing different types of slightly more complex processes, some service users needed help from family members.

##### Provides a Sense of Privacy

The service users in the study felt safe for the most part in the space that was provided for their eHealth activity and valued the direct contact it afforded with caregivers. When weighing the risk that an unauthorized person would get access to personal health-related information against the help they get via eHealth, they considered it worthwhile. This approach to privacy risks allowed participants to use eHealth; however, they also mentioned that the 2-factor authentication enabled their sense of privacy.

##### Personal Feedback and Staff Support

Service users perceived that personal contact with health care professionals before and during the eHealth intervention was an important facilitator. The study participants described a need for much information before the actual intervention began; they wanted information about the content of the eHealth program and also how to manage the computer-related tasks involved. Individual contact with health care personnel during ongoing interventions was perceived as important and even necessary. As eHealth was perceived as mostly managing health problems on one’s own, being provided with an option to call someone for support was very valuable.

##### Flexible Use of Time

Many study participants perceived that their eHealth intervention took less time compared with, for example, physical visits within the care system. Among other things, service users appreciated that they did not have to set aside time for travel or look for parking and that participating in an eHealth intervention was easier than attending a traditional treatment with physical meetings as it was easier to fit into their daily schedule.

Service users perceived a greater degree of self-determination and awareness about when and how they conducted the treatment and appreciated feelings of being in control. For example, they could do exercises whenever they wanted and could read at their own pace.

##### A Credible Sender

Service users expressed that they perceived the sender of an eHealth intervention as reliable and credible, which was an implementation facilitator from their perspective. Service users described that they needed to feel that they could trust the organization *behind* the intervention. Public actors such as universities and public health care organizations were given greater credibility than private actors. Service users also described that it was important that they knew or experienced trust for the person responsible for their treatment and needed to know that they could get in touch with that person if needed.

### Differences and Commonalities

#### Overview

In this section, we present the results of our supplementary analysis (Multimedia Appendix 1). In Multimedia Appendix 1, the boxes indicate subcategories from step 1, sorted by stakeholder group (policy makers and service users) and main category (barriers and facilitators). Arrows indicate similarities between stakeholder group perceptions, as expressed in the subcategories. The differences between the stakeholder subcategories are not marked.

#### Barriers

Policy makers perceived that a barrier to the implementation of eHealth was that not all service users benefit from eHealth. This was mirrored in the perceptions of service users, who highlighted the individual ability and preferences of service users as potential barriers.

Although policy makers perceived that there were implementation barriers in their work role (the complexity of decision-making, a lack of resources for health care digitalization, and fear of technical infrastructure problems), service users expressed that the design of the technology needed to be matched to their needs and that a perceived lack of data protection could be an implementation barrier to service users.

Both groups reflected on a possible redistribution of *work burden* between professionals and service users. Policy makers expressed some uncertainty about the work consequences for health care professionals, and some service users perceived that they found themselves doing more of the professionals’ work.

#### Facilitators

Policy makers and service users all perceived that the large impetus toward the implementation of eHealth should be considered an important facilitator. Policy makers were convinced about the public *pull* toward eHealth and believed that the importance of their policy mission would facilitate implementation.

Service users were more specific in describing which actual eHealth features of the technology would facilitate the implementation; not only should the eHealth design be user friendly and provide flexible use of time but also that it should provide a sense of privacy and a credible sender behind the technology.

Although policy makers believed that health care practice would benefit from eHealth by freeing up staff hours and saw that as an implementation driver, service users saw personal feedback and support from health care staff as an implementation facilitator.

## Discussion

### Principal Findings

This study shows that perceptions of policy makers and service users tend to converge concerning the perception of general societal impetus toward the adoption of eHealth and the promise of effectiveness through the flexibility of time use. The 2 groups also agreed that the implementation of eHealth may be hindered by the difficulties in tailoring eHealth to user differentiation and expressed that an implementation barrier could be the uncertainty about the ultimate distribution of care burden. The perceptions of policy makers and service users did not converge in other aspects. At the back end of implementation, policy makers perceived decision-making as complex, as technical and regulatory systems are lacking in relevance and detail, and knowledge and resources for implementation (ie, for building the eHealth infrastructure) are scarce. At the front end, service users highlighted the importance of the protection of digital data and trust in the sender.

In the following sections, we will use the domains in the *nonadoption, abandonment, spread, scale up, and sustainability* framework (framework for considering influences on the adoption, nonadoption, abandonment, spread, scale up, and sustainability of patient-facing health and care technologies) [8] to frame our discussion of the commonalities and differences in the perceived barriers to and facilitators of the implementation of eHealth between policy makers and service users.

In our study, both groups appeared to agree that the *condition and illness* of the service user is a factor to consider. They identified a common barrier in that eHealth is not a suitable or sufficient way of providing health care to every member of the general population. Although policy makers mainly focused on criteria such as high age and critical health as barriers, service users also identified the timing of eHealth interventions in their disease progress and their individual expectations of efficacy. The implementation of digital technology will always be more complex when the condition is poorly characterized, poorly understood, unpredictable, or high risk [8], and studies have shown that the medical and sociocultural criteria for patient selection for eHealth interventions are still in its infancy in many instances [22,23]. Greenhalgh et al [8] also reported that only a fraction of potential end users was assessed by their clinicians as *suitable* for an eHealth intervention. In the SARS-CoV-2 pandemic era, the use of digital tools has accelerated and spread more widely to many new areas of health care [24-26]. The question of whether current expectations about the general applicability of eHealth are, in fact, realistic remains to be seen. Recent publications note that despite societal pressures because of the pandemic, there is no extensive clinical experience of eHealth in cardiac care and highlight both the limitations and usefulness of digital solutions for managing CVD [27,28].

The *technology* itself has a prominent role in the perceptions of both groups. Service users expressed that the feeling of being in a digitally protected environment and trusting the *sender* facilitated implementation. Other research has confirmed that the authority of the author has been found to influence trust and credibility [29,30]. Although the policy makers in the study perceived that most of their citizens were interested in digital health innovation, they did not elaborate on the importance of cybersecurity and expressed that they had limited knowledge about the key features of different types of eHealth. Wozney et al [31] interviewed key informants who were influential in the adoption of technology for e-Mental health and found similar knowledge gaps. Swedish national laws and European laws are very clear about the responsibilities of the government and policy makers to protect citizens from harm through health data breaches [32,33]. In view of the need to accelerate the implementation of eHealth, Swedish authorities have recently devised an action plan. The main action targets in the plan are more focused attention to issues of data protection, the involvement of individual service users and health care staff, provision of information and knowledge, and promotion of collaboration around digital transformation [34]. A recent review focusing on the implementation of videoconferences in diverse areas of health care similarly found several challenges related to service development [35].

Notwithstanding the limited scientific evidence of the effectiveness of eHealth, policy makers and service users alike perceived that eHealth can enable more effective use of time in the management of health-related problems as a *value proposition*. However, one of the findings of the study was that both groups expressed uncertainty about the distribution of care burden. Service users saw eHealth as a way of making health care fit in their daily schedule and meeting their needs. Other studies confirm these perceptions of benefits for service users [12,36-38]. Our study showed that service users could also perceive their eHealth intervention as time consuming; however, they expressed concern about the shifting of roles and responsibilities (and workload) from health care practitioners to service users and their caregivers. This shift in the burden of care has been reported to change the work required of patients and be a potential barrier for the use of eHealth, which may lead to nonuse by service users [8,39]. Policy makers primarily saw eHealth as potentially beneficial for health care practice, for example, a way of reducing care tasks among health care professionals. In a recent systematic review of primary health care workers’ perceptions and experiences of mobile health (mobile technology and communication tools such as smartphones, tablets, PDAs, and wearable devices such as smartwatches for the prevention, promotion, treatment, and maintenance of health, information, and data collection), results were shown to be rather more mixed. Although health workers recognized some time benefits, they saw mobile health as slow and time consuming in some cases and as creating more work [40]. The findings of Greenhalgh et al [8] point to the real risk of some staff simply not engaging with the program or using the technology. In a recent review by Drissi et al [41], only a small part of the included studies provided an empirical evaluation of the reported digital interventions to assist health care workers in mental care, and half of the studies listed challenges and limitations related to the adoption of the reported interventions.

Policy makers and service users in the study both agreed that eHealth potentially allows patients to be more empowered to participate in their own care. Similarly, a study by Whittaker [42] showed that policy makers, administrators, and organizational leaders in the United States viewed eHealth technology as potentially transformative for health care. Other research shows that patient-facing technologies may not only inform, educate, and empower but also sometimes be misinterpreted and cause distress [8,43]. In this study, service users expressed concerns that implementing eHealth would mean less human contact with health care professionals, which is something they saw as an important quality marker for health care. A recent systematic review found that there were few studies that identified ethical issues associated with telehealth practice [44]. A systematic review by Parker et al [45] found that differences in socioeconomic factors and gender were associated with the use of remote consultations and internet-based consultations in general practice. In their systematic review of the use of eHealth technology in cardiac care, Harky et al [46] highlighted the need for further studies exploring how staff–service user communications answer the call for service user empowerment.

The 2 stakeholder groups differed in their perceptions of factors related to *adopters.* One of the important results from our analysis was that policy makers did not explicitly show an awareness of the features of eHealth that service users perceived as important facilitators of their engagement with the technology. Although policy makers focused on the back-end challenges with eHealth, for example, the difficulty of providing regional broadband coverage, service users were mostly concerned about the interface and their own ability to work, read, and use the digital solution. Research has shown that the information quality and the demands of the technology on service users’ computer literacy skills and other individual competencies are very important [47,48]. However, tailored solutions that cover variable competencies come at a considerable monetary cost [8]. As the policy makers in the study expressed the real-life need to align their implementation decisions to a health care system with a nonexpanding budget, individual tailoring of technologies to differing needs will realistically be limited.

In an *organizational and wider system* context, the policy makers in the study had an ambition toward making their region’s digitalization compare favorably with other parts of the country. This raises the question of which priorities will be set in the near future: regional interests or collaboration across regional borders. Policy makers perceived uncertainty in their decision-making, as there was a lack of regulatory support to guide their decision-making and no extra resources for implementing the technologies. The Swedish vision of national digitalization of health care by the year 2025 [5] stands in stark contrast to policy makers’ perceptions of decisional difficulties, pointing to the need for more coherent national planning, strategic reimbursement, and regulatory support. Other research also reports key barriers in micro- and mesolevel contexts of health care organizations; however, more research is needed that focuses explicitly on policy development and implementation planning [29,49,50].

Neither policy makers nor service users reflected on the *embedding of technology in health care systems* and the *adaptation of technology over time.* Although the research literature on the use of eHealth has exploded during the SARS-CoV-2 pandemic, not many studies have used more rigorous theoretical tools to study the long-term issues of using eHealth technologies.

The World Health Organization highlights that there is a need to go beyond the small-scale implementation of pilot projects focusing on the evidence of feasibility and effect to a more extensive exploration of the infrastructure needed to scale up and sustain digital health technology as a global issue in the future. *Scaling up* comprises deliberate efforts to increase the impact of innovations that were successfully tested in pilot or experimental projects so as to benefit more people. Policy and program development on a lasting basis may be fostered through government adoption, commercial adoption, or hybrid models [51]. Stable and secure financial and technical resources and enduring partnerships are the foundation for sustainability, in addition to the capacity to continually adapt the product to meet the demands of service users and the ever-changing operational environment [52]. However, an understanding building on new insights may take some time. Greenhalgh et al [8] proposed that some of the barriers to achieving better eHealth implementation may involve multiple strategies at the organizational and societal level, including a revision of laws and a gradual “shared sense-making through reflexive monitoring and analysis of critical events or issues”

In any event, outcomes from eHealth solutions need reasonably be superior or at least comparable with traditional health care practices in terms of safety, timeliness, equity, effectiveness, efficiency, and patient centeredness. The SARS-CoV-2 pandemic has made practitioners and researchers more aware that although eHealth solutions have a great attraction, there are many aspects that remain to be explored. New research findings will likely fuel the discussion on the digital transformation of society and health care. Understanding the complexities involved in this transformation is a challenge not only for the public but also for policy makers at all levels; setting an even more proactive agenda for the solid and high-quality implementation of eHealth will be necessary. To make well-based policy decisions concerning the implementation of eHealth, policy makers will need to be informed by research that takes the complexity of implementation into account and pays more systematic attention to implementation outcomes that ensure the quality of care.

Before attempting to implement various eHealth solutions, implementation studies are not only needed to identify and address numerous barriers to implementation and understand how interventions can be sustainably translated from research into clinical practice but also to evaluate outcomes with standardized evaluation methods and instruments that are lacking at present [53].

### Strengths and Limitations of the Study

As the study was conducted in prepandemic conditions, the incentive to use eHealth was not as strong as it has since become, and perceptions about using eHealth technology will likely have evolved in both stakeholder groups.

The participants in both the policy maker and the service user group had a variety of demographic characteristics, which indicates some representativeness. The perspectives of policy makers, who are an underrepresented group in health care research, contribute to a broader insight into barriers to and facilitators of the implementation of eHealth. The scope of the service users’ perceptions of eHealth implementation, in general, was possibly somewhat limited by the fact that the focus in the interviews was on their experiences with the specific eHealth intervention (e-Mental health in cardiac care) in which they were enrolled. However, as the service users actively participated in eHealth interventions, they were more knowledgeable about eHealth than other members of the public, which benefited the study.

The authors’ group expertise, with competencies in implementation science, extensive clinical experience in health care, and experience of qualitative methodology, contributes to methodological trustworthiness.

### Conclusions and Clinical Implications

This study provides previously unavailable information about key informant perspectives on eHealth implementation. The study not only shows that both policy makers and service users perceive an impetus toward the implementation of eHealth but also that there are differences in views concerning implementation challenges and that policy makers do not perceive the barriers and facilitators in the same way as service users do. Dissonant perceptions about a *new distribution of workload* emerged; although policy makers see eHealth as potentially freeing up staff hours, service users highlight their need for data security, feedback, and support and may sometimes even see eHealth as the lesser alternative.

To be able to gear policy making to match service user needs and preferences, future research should explore avenues toward a more effective knowledge exchange regarding eHealth implementation between different stakeholder groups and systematic use of implementation science frameworks. Further research is needed to clarify the perspectives of health care staff and their clinical leaders on the sustainable implementation of eHealth in clinical practice and how their perceptions overlap with the views expressed by the stakeholders in this paper.

## Appendix

Multimedia Appendix 1 Visual illustration of the qualitative content analyses.

## Abbreviations

CVD: cardiovascular disease
iCBT: internet-based cognitive behavioral therapy
HeaDING: Heart Failure and Depression Using Internet Based–Cognitive Behavioural Therapy

## References

[1] (2019). State of Health in the EU: shift to prevention and primary care is the most important trend across countries. European Commission.

[2] World Health Organization (2016). Global Diffusion of eHealth: Making Universal Health Coverage Achievable: Report of the Third Global Survey on eHealth.

[3] Black AD, Car J, Pagliari C, Anandan C, Cresswell K, Bokun T, McKinstry B, Procter R, Majeed A, Sheikh A (2011). The impact of eHealth on the quality and safety of health care: a systematic overview. PLoS Med.

[4] Flodgren G, Rachas A, Farmer AJ, Inzitari M, Shepperd S (2015). Interactive telemedicine: effects on professional practice and health care outcomes. Cochrane Database Syst Rev.

[5] Swedish Government and Swedish Association of Local Authorities and Regions (SALAR) (2020). Vision for eHealth 2025 : follow-up 2019. E-Hälsa 2025.

[6] Mair FS, May C, O'Donnell C, Finch T, Sullivan F, Murray E (2012). Factors that promote or inhibit the implementation of e-health systems: an explanatory systematic review. Bull World Health Organ.

[7] Ross J, Stevenson F, Lau R, Murray E (2016). Factors that influence the implementation of e-health: a systematic review of systematic reviews (an update). Implement Sci.

[8] Greenhalgh T, Wherton J, Papoutsi C, Lynch J, Hughes G, A'Court C, Hinder S, Fahy N, Procter R, Shaw S (2017). Beyond adoption: a new framework for theorizing and evaluating nonadoption, abandonment, and challenges to the scale-up, spread, and sustainability of health and care technologies. J Med Internet Res.

[9] Glouberman S, Mintzberg H (2001). Managing the care of health and the cure of disease - Part I: Differentiation. Ovid.

[10] O'Connor S, Hanlon P, O'Donnell CA, Garcia S, Glanville J, Mair FS (2016). Understanding factors affecting patient and public engagement and recruitment to digital health interventions: a systematic review of qualitative studies. BMC Med Inform Decis Mak.

[11] Swinkels IC, Huygens MW, Schoenmakers TM, Nijeweme-D'Hollosy WO, van Velsen L, Vermeulen J, Schoone-Harmsen M, Jansen YJ, van Schayck OC, Friele R, de Witte L (2018). Lessons learned from a living lab on the broad adoption of eHealth in primary health care. J Med Internet Res.

[12] Slater H, Campbell JM, Stinson JN, Burley MM, Briggs AM (2017). End user and implementer experiences of mhealth technologies for noncommunicable chronic disease management in young adults: systematic review. J Med Internet Res.

[13] Damschroder LJ, Aron DC, Keith RE, Kirsh SR, Alexander JA, Lowery JC (2009). Fostering implementation of health services research findings into practice: a consolidated framework for advancing implementation science. Implement Sci.

[14] Vassilev I, Rowsell A, Pope C, Kennedy A, O'Cathain A, Salisbury C, Rogers A (2015). Assessing the implementability of telehealth interventions for self-management support: a realist review. Implement Sci.

[15] Varsi C, Nes LS, Kristjansdottir OB, Kelders SM, Stenberg U, Zangi HA, Børøsund E, Weiss KE, Stubhaug A, Asbjørnsen RA, Westeng M, Ødegaard M, Eide H (2019). Implementation strategies to enhance the implementation of eHealth programs for patients with chronic illnesses: realist systematic review. J Med Internet Res.

[16] Lundgren JG, Dahlström O, Andersson G, Jaarsma T, Kärner KA, Johansson P (2016). The effect of guided web-based cognitive behavioral therapy on patients with depressive symptoms and heart failure: a pilot randomized controlled trial. J Med Internet Res.

[17] Johansson P, Westas M, Andersson G, Alehagen U, Broström A, Jaarsma T, Mourad G, Lundgren J (2019). An internet-based cognitive behavioral therapy program adapted to patients with cardiovascular disease and depression: randomized controlled trial. JMIR Ment Health.

[18] Siebmanns S, Johansson P, Ulander M, Johansson L, Andersson G, Broström A (2021). The effect of nurse-led internet-based cognitive behavioural therapy for insomnia on patients with cardiovascular disease: a randomized controlled trial with 6-month follow-up. Nurs Open.

[19] Eccles MP, Mittman BS (2006). Welcome to Implementation Science. Implementation Sci.

[20] Heaton J (2008). Secondary analysis of qualitative data: an overview. Histor Soc Res / Historische Sozialforschung.

[21] Hsieh H, Shannon SE (2005). Three approaches to qualitative content analysis. Qual Health Res.

[22] Joseph V, West RM, Shickle D, Keen J, Clamp S (2011). Key challenges in the development and implementation of telehealth projects. J Telemed Telecare.

[23] Paré G, Moqadem K, Pineau G, St-Hilaire C (2010). Clinical effects of home telemonitoring in the context of diabetes, asthma, heart failure and hypertension: a systematic review. J Med Internet Res.

[24] Hincapié MA, Gallego JC, Gempeler A, Piñeros JA, Nasner D, Escobar MF (2020). Implementation and usefulness of telemedicine during the COVID-19 pandemic: a scoping review. J Prim Care Community Health.

[25] Lieneck C, Weaver E, Maryon T (2021). Outpatient telehealth implementation in the united states during the COVID-19 global pandemic: a systematic review. Medicina (Kaunas).

[26] Gunasekeran DV, Tseng RM, Tham Y, Wong TY (2021). Applications of digital health for public health responses to COVID-19: a systematic scoping review of artificial intelligence, telehealth and related technologies. NPJ Digit Med.

[27] Kaushik KA, Patel S, Dubey K (2020). Digital cardiovascular care in COVID-19 pandemic: a potential alternative?. J Card Surg.

[28] Ajibade A, Younas H, Pullan M, Harky A (2020). Telemedicine in cardiovascular surgery during COVID-19 pandemic: a systematic review and our experience. J Card Surg.

[29] Sbaffi L, Rowley J (2017). Trust and credibility in web-based health information: a review and agenda for future research. J Med Internet Res.

[30] Westas M, Lundgren J, Mourad G, Neher M, Johansson P (2020). How healthcare professionals in cardiac care address depressive symptoms: experiences of patients with cardiovascular disease. J Cardiovasc Nurs.

[31] Wozney L, Newton AS, Gehring ND, Bennett K, Huguet A, Hartling L, Dyson MP, McGrath P (2017). Implementation of eMental Health care: viewpoints from key informants from organizations and agencies with eHealth mandates. BMC Med Inform Decis Mak.

[32] (2016). Regulation (EU) 2016/679 of the European Parliament and of the Council. European Union - General Data Protection Regulation.

[33] (2020). Patient Safety Act (2010: 659). Swedish Patient Safety Law.

[34] Swedish Government and Swedish Association of Local Authorities and Regions (SALAR) (2021). Genomförandeplan 2020–2022 Bilaga till Strategidokument Vision e-hälsa 2025. E-Hälsa 2025.

[35] James HM, Papoutsi C, Wherton J, Greenhalgh T, Shaw SE (2021). Spread, scale-up, and sustainability of video consulting in health care: systematic review and synthesis guided by the NASSS framework. J Med Internet Res.

[36] Struthers A, Charette C, Bapuji SB, Winters S, Ye X, Metge C, Kreindler S, Raynard M, Lemaire J, Synyshyn M, Sutherland K (2015). The acceptability of e-mental health services for children, adolescents, and young adults: a systematic search and review. Can J Commun Ment Health.

[37] Thiyagarajan A, Grant C, Griffiths F, Atherton H (2020). Exploring patients' and clinicians' experiences of video consultations in primary care: a systematic scoping review. BJGP Open.

[38] Tully L, Case L, Arthurs N, Sorensen J, Marcin JP, O'Malley G (2021). Barriers and facilitators for implementing paediatric telemedicine: rapid review of user perspectives. Front Pediatr.

[39] Gilbert A, Jones J, Jaggi A, May C (2020). Use of virtual consultations in an orthopaedic rehabilitation setting: how do changes in the work of being a patient influence patient preferences? A systematic review and qualitative synthesis. BMJ Open.

[40] Odendaal WA, Watkins JA, Leon N, Goudge J, Griffiths F, Tomlinson M, Daniels K (2020). Health workers' perceptions and experiences of using mHealth technologies to deliver primary healthcare services: a qualitative evidence synthesis. Cochrane Database Syst Rev.

[41] Drissi N, Ouhbi S, Marques G, de la Torre Díez I, Ghogho M, Idrissi MA (2020). A systematic literature review on e-mental health solutions to assist health care workers during COVID-19. Telemed J E Health.

[42] Whittaker R (2012). Issues in mHealth: findings from key informant interviews. J Med Internet Res.

[43] de Vries AE, van der Wal MH, Nieuwenhuis MM, de Jong RM, van Dijk RB, Jaarsma T, Hillege HL (2013). Health professionals' expectations versus experiences of internet-based telemonitoring: survey among heart failure clinics. J Med Internet Res.

[44] Keenan AJ, Tsourtos G, Tieman J (2021). The value of applying ethical principles in telehealth practices: systematic review. J Med Internet Res.

[45] Parker RF, Figures EL, Paddison CA, Matheson JI, Blane DN, Ford JA (2021). Inequalities in general practice remote consultations: a systematic review. BJGP Open.

[46] Harky A, Adan A, Mohamed M, Elmi A, Theologou T (2020). Technology and cardiovascular diseases in the era of COVID-19. J Card Surg.

[47] Su JJ, Yu DS, Paguio JT (2020). Effect of eHealth cardiac rehabilitation on health outcomes of coronary heart disease patients: a systematic review and meta-analysis. J Adv Nurs.

[48] Baumel A, Kane JM (2018). Examining predictors of real-world user engagement with self-guided ehealth interventions: analysis of mobile apps and websites using a novel dataset. J Med Internet Res.

[49] Batterham PJ, Sunderland M, Calear AL, Davey CG, Christensen H, Teesson M, Kay-Lambkin F, Andrews G, Mitchell PB, Herrman H, Butow PN, Krouskos D (2015). Developing a roadmap for the translation of e-mental health services for depression. Aust N Z J Psychiatry.

[50] Meurk C, Leung J, Hall W, Head BW, Whiteford H (2016). Establishing and governing e-mental health care in australia: a systematic review of challenges and a call for policy-focussed research. J Med Internet Res.

[51] World Health Organization (2015). The MAPS Toolkit: mHealth Assessment and Planning for Scale.

[52] Simmons R, Fajans P, Ghiron L, Simmons R, Fajans P, Ghiron L (2007). Introduction. Scaling Up Health Service Delivery: From Pilot Innovations to Policies and Programmes.

[53] Vis C, Bührmann L, Riper H, Ossebaard HC (2020). Health technology assessment frameworks for eHealth: a systematic review. Int J Technol Assess Health Care.

