# Supplementary figures and images for: Perspectives of Policy Makers and Service Users Concerning the Implementation of eHealth in Sweden: Interview Study
Source: J Med Internet Res. 2022 Jan 28;24(1):e28870. doi: 10.2196/28870 (PMC8838545; doi:10.2196/28870)

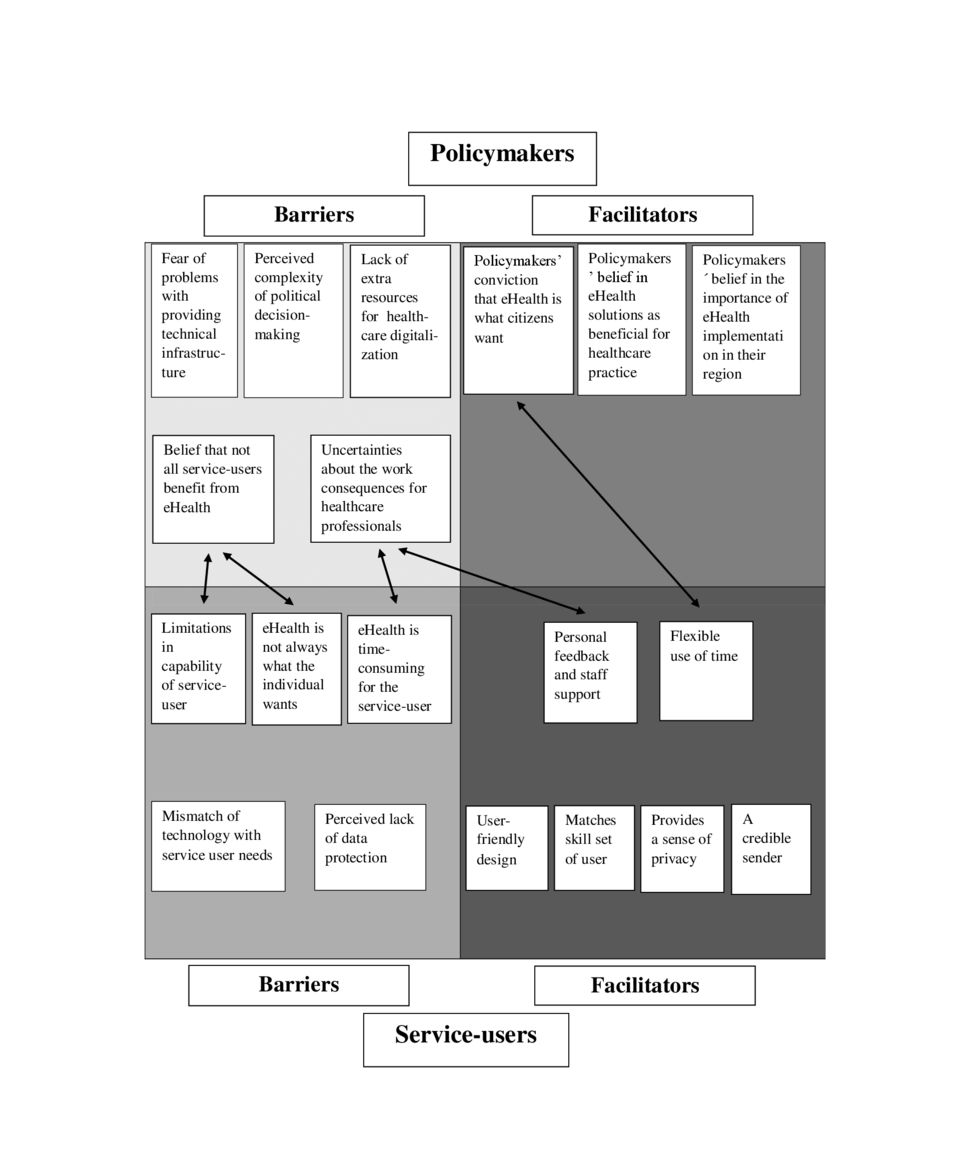

Supplement: Multimedia Appendix 1 [file jmir_v24i1e28870_app1.png]
